# Supplementary material for: Characterization, Genome Sequencing, and Development of a Rapid PCR Identification Primer for Fusarium oxysporum f. sp. crocus, a New forma specialis Causing Saffron Corm Rot
Source: Plants (Basel). 2024 Nov 11;13(22):3166. doi: 10.3390/plants13223166 (PMC11597565; doi:10.3390/plants13223166)
Supplement: Supplementary file 1 [file plants-13-03166-s001.zip › plants-3247935-supplementary.pdf]

Supplementary Table S1. Information on all strains used in this study

1.1 Information on the strains used in this study for the construction of the gene evolution tree

Gene	Host	Formae speciale	GenBank	Reference
SIX 4	*C. sativus*	*crocus*	PP855472	This study
	S. lycopersicum	*lycopersici*	GQ268951	Inami et al. (2012)
	M. nana	*cubense*	KX435004	Czislowski et al. (2018)
			KX435005	
			KX435006	
SIX 6	*C. sativus*	*crocus*	PP857836	This study
	M. nana	*cubense*	KX435008	Czislowski et al. (2018)
	Gossypium	*vasinfectum*	KR855787	Rocha et al. 2016
	S. lycopersicum	*lycopersici*	KR855786	
	P. vulgaris	*phaseoli*	KP681651	
	P. sativum	*pisi*	KR855779	
	C. sativus	*cucumerinum*	KR855770	
	C. lanatus	*niveum*	KR855756	
	Passiflora	*passiflorae*	KR855782	
SIX 7	*C. sativus*	*crocus*	PP885435	This study
	S. lycopersicum	*lycopersici*	GQ268954	Lievens et al. (2009)
	P. canariensis	*canariensis*	KM893939	
	P. sativum	*pisi*	KP964969	Taylor et al. (2016)
	Narcissus	*narcissi*	KP964972	
	Lilium	*lilii*	GQ268960	Lievens et al. (2009)
	Zingiberi	*zingiberi*	KX435050	Czislowski et al. (2018)

	M. nana	*cubense*	KX435010	
	P. vulgaris	*phaseoli*	MG680406	Simbaqueba et al. (2018)
SIX 10	*C. sativus*	*crocus*	PP857840	This study
	P. canariensis	*canariensis*	KM893959	Laurence et al. (2015)
	L. Sesamum	*sesami*	MN417217	Duan et al. (2020)
	S. lycopersicum	*lycopersici*	KC701448	Schmidt et al. (2013)
	Physalis	*physali*	MG680407	Simbaqueba et al. (2018)
	Zingiberi	*zingiberi*	KX435051	Czislowski et al. (2018)
	M. nana	*cubense*	KX435018	
	Narcissus	*narcissi*	KP964986	Taylor et al. (2016)
	A. cepa	*cepae*	KP964987	
	P. sativum	*pisi*	KP964983	
	Linum	*lini*	KP964982	
	F. hybrida klatt	*freesia*	KP964984	
SIX 11	*C. sativus*	*crocus*	PP857837	This study
	S. lycopersicum	*lycopersici*	KC701449	Schmidt et al. (2013)
	Passiflora	*passiflorae*	KX435049	Czislowski et al. (2018)
	C. lanatus	*niveum*	KX435042	
	P. sativum	*pisi*	KP964988	Taylor et al. (2016)
	P. vulgaris	*phaseoli*	KP964989	
SIX 12	*C. sativus*	*crocus*	PP857839	This study
	P. canariensis	*canariensis*	MH746920	-
	S. lycopersicum	*lycopersici*	KC701450	Schmidt et al. (2013)
	Zingiberi	*zingiberi*	KX435052	Czislowski et al. (2018)

	A. cepa	*cepae*	KP964996	Taylor et al. (2016)
	P. sativum	*pisi*	KP964993	
	F. hybrida klatt	*freesia*	KP964994	
	Narcissus	*narcissi*	KP964990	
	Linum	*lini*	KP964992	
SIX 14	*C. sativus*	*crocus*	PP857838	This study
	S. lycopersicum	*lycopersici*	KC701452	Schmidt et al. (2013)
	F. hybrida klatt	*freesia*	KP965002	Taylor et al. (2016)
	P. sativum	*pisi*	KP965003	
			KP965004	
			KP965005	
	A. cepa	*cepae*	KP965006	

1.2 Information on saffron and other strains

Host	species	Strain number	Reference
saffron	*F. oxysporum*	XHH2	Ren et al. (2021)
		XHH16	
		XHH35	
		XHH47	
		XHH56	
		XHH66	
		XHH67	
		XHH76	
		XHH78	
		XHH80	
tomato	*F. oxysporum* f. sp. *lycopersici*	FQ-1	Laboratory separation and preservation
watermelon	*F. oxysporum* f. sp. *niveum*	XG-3	
melon	*F. oxysporum* f. sp. *melonis*	TG-7	
strawberry	*F. oxysporum* f. sp. *fragariae*	H80	
–	*F. fujikuroi*	Ff-3	
	Colletotrichum	Co-5	
	Alternaria alernata	Aa-1	
	Botryosphaeria dothidea	Bd-1	
	Corynespora	Cor-1	
	Nigrospora	Ni-1	

Supplementary Table S2. *SIX* gene primers for identification of specialized *F. oxysporum* of saffron corm rot

Gene	Primers	Sequence 5'-3'	Product size (bp)	Annealing temperature (℃)	Reference
SIX 1	SIX1-F	TCTCCATTACTTTGTCTCACG	694–733	58	Czislowski *et al.* (2021)
	SIX1-R	CGATTTAGGCGATTCGGGG			
SIX 2	SIX2-F	GGTTCCCATCGTTGAAGC	327–330	57	Czislowski *et al.* (2021)
	SIX2-R	TTGGTTTAAATCTGCGTGTC			
SIX 3	SIX3-F	TTACTACGAGCTTCAGCACC	223	60	Czislowski *et al.* (2021)
	SIX3-R	GCATTAGGTGTTGCAACAGG			
SIX 4	SIX4-F	GAACCTGACGTTACTTGCGAT	344	58	This study
	SIX4-R	GCTAAGTTAATTGTACCTTG			
SIX 5	SIX5-F	TCATCAGTACTGTGCTTGCC	347–354	59	Czislowski *et al.* (2021)
	SIX5-R	CATGTTGAGTCTGCTCCTCC			
SIX 6	SIX6-F	CGAGCACACCCTTCACTATG	509	58	This study
	SIX6-R	CTGAATAGGTCACTACTGAAG			
SIX 7	SIX7-F	GAGGTGACATTTGACATCACC	113	60	This study
	SIX7-R	TAGTATGCGCGCCATTGG			
SIX 8	SIX8-F	CCCTAGCCGTCTCTGTGGC	163–165	64	Czislowski *et al.* (2021)
	SIX8-R	CGTTCGACAAGGGCTCTCTCG			
SIX 9	SIX9-F	CCGTCTTCTCTACCGCCG	288	58	Czislowski *et al.* (2021)
	SIX9-R	AGTTGACGCAAGCAAAGTCG			
SIX 10	SIX10-F	GTTAGCAACTGCGAGACACTAGAA	636	65	Taylor *et al.* (2016)
	SIX10-R	AGCAACTTCCTTCCTCTTACTAGC			

SIX 11	SIX11-F	GTTGCTCCTCCTTTGCTGG	163	62	This study
	SIX11-R	TACCACTCTGACCAGTCACC			
SIX 12	SIX12-F	CTAACGAAGTGAAAAGAAGTCCTC	449	61	Taylor *et al.* (2016)
	SIX12-R	GCCTCGCTGGCAAGTATTTGTT			
SIX 13	SIX13-F	CCTTCATCATCGACAGTACAACG	527	61	Czislowski *et al.* (2021)
	SIX13-R	ATCAAACCCGTAACTCAGCTCC			
SIX 14	SIX14-F	ATAAAGTGCGACTGGGACTTCTGC	422	67	Taylor *et al.* (2016)
	SIX14-R	ACCCCCATCCACATTCCTAAGCGA			

Supplementary Table S3. Genbank accessions of the *SIX* gene homologues from *Fusarium oxysporum* f. sp. *lycopersici* (*Fol*) used for query sequences to seach the BLAST nucleotide database for *SIX* gene orthologues.

SIX 1	***SIX 2***	***SIX 3***	***SIX 4***	***SIX 5***	***SIX 6***	***SIX 7***
XM_018396441.1	XM_018396439.1	XM_018396424.1	GQ268951.1	GQ268952.1	XM_018394324.1	FJ755836.1
SIX 8	***SIX 9***	***SIX 10***	***SIX 11***	***SIX 12***	***SIX 13***	***SIX 14***
FJ755837.1	XM_018394292.1	XM_018397464.1	KC701449.1	KC701450.1	XM_018397144.1	KC701452.1

Supplementary Table S4. Seven *SIX* genes highly homologous to *Fol* were identified in the *F. oxysporum* saffron strain (XHH35)

Species	Gene	Sequences	Homology	E-value	q_len	align_len
F. oxysporum saffron strain (XHH35)	*SIX 4*	Sequences 1	79.058	8.99E-96	242	191
	SIX 6	Sequences 2	53.333	4.19E-62	225	206
	SIX 7	Sequences 3	68.992	1.49E-43	220	129
	SIX 10	Sequences 4	86.726	3.13E-53	241	113
	SIX 11	Sequences 5	94.595	7.81E-68	110	110
	SIX 12	Sequences 6	88.496	1.52E-62	113	113
	SIX 14	Sequences 7	90	2.73E-31	88	40

SIX 4 (Sequences 1):<br>GCGGGAATCTCGTTAAAAGATCTGCTTTGACCCAATCCGTTTGTACGCATGCTGGCGCTGCTACTGGCACAGATCTCTATTGGATCGCGGAATAT<br>GCACTGGTCAATCGACATATACTGTTAACTGCGCGCCTGCGGATAACAAAAATGCTGGATCGTCACACCCTGGGACCTGTCCCTCAGGTGAAGAT<br>TGCTTCCAGTTAGAGCGAGTTGGCAACTTTTGGGGTGATATGGAACCTGACGTTACTTGCGATAAATCCGGCACTGTTTTGATGCGGCAGATGTC<br>AAGACGGCCACGCACGTAAATGGCAAGGTTGTCACGCGTGCTGGAAAGCCAAATATACAACCAAGGCTCATTCGACTCAAGGCCCAAGTCTACA<br>GCCGGGACGGGCACTATGGTCAAACTTCGAGAATGGGCTTTTTCGCAATGACGTGGAGCAATACGGCATCAATGATGTGGCAAGTATGGGACCG<br>ACGTGGAACTTCGACCCTTCCGTGGACGAGAGCTTTAGCTTCTTCTTCACTCCAGGGCCCAATGCATTTCGCATTCAAGGTACAATTAACTTAGCT
SIX 6 (Sequences 2):<br>TGAAGGTCGCTCTCGTTATATCAATCTTTATTGCTAGCTGCATTGCTAGTCCCCTGGACCCAGCTAAAACACCTACGTCACCGCCTGGGGCC<br>GAGCACACCCTTCACTATGTTGATGAAGGTCCCACGGGGCCTCCATTCTTCCCCGTCGATAATTCGTCAACCTTGGTCTCTCGAGACACGCT<br>TCCCCCGACCTGTCCAACCGGCACGACATATGACAGCTCTACTTGCTTCAATTCCAGCTTCATTCGTGGCCTTTGCGTCTCGAACCGCCCGG<br>GAGGCCTGGGGGTTCGCACTGGTGCTAATTGCAACAGAAACGAGATATGCGTACAGCGCAACCTTAGCAACGGAAAACCCTATGCTAAAT<br>GCATTGATATTCACCAACTTGTCTCGTGGAAAACTTCCCCGGACGGGGATAAATCAGGCTGCACTACAGTCGAAGCCAGTCCTATAGGATC<br>TTATAAGTTGGGCACAATTGTATATGACGTCAACAAACACCCTATTCAAGTTTCTAAGATTAATTACCTTGGAGAGCCTGGCGATGCAGAT

GATGGTATTGGAGGATCAGTATCATCCTTCAGTAGTGACCTATTCAGGTTCACAGGATCTAATTATATGAAA
SIX 7 (Sequences 3):<br>ATGCAGATTATGAAGTACCTTTACCTCCTTTTCCATTTCGCCCTGTTTGCGAGTGCTATACCTACACTCGATCTCTTTCCAAGGCAAGGGCAA<br>TGCCTTACCACCCGCTCAAGAGCAGCAGGACCACCGGAAACGGCTCCCGAGGTGACATTTGACATCACCGAAAATGTCGACAGGTTCGCC<br>AGCGCCGCGTCTTCACCATGGACTCAGGGTGTAGGAATCCCGAATGTTAGATACCAATGGCGCGCATACTACAGTCTTAGAGCTCAAACTA<br>CATTCGTAGACATTCAGGTTTTCGGTACTGCCAGCGTACAAGCAATCTTGCTTCCGGATGCTCCCTCAGCTGGTCATTGGCACGCTATCGAT<br>AGCACCTTAGTGAGGCC
SIX 10 (Sequences 4):<br>CTAAACGAGATGTACCATACAGATTTGATATCACGTTTCGAGTTGGTCCAGCCGGGGCTAGCGTCGCGCCATTCTCTGGATCTGTGTACGTC<br>CTGGATGGTGTTACCCCACTTGTTCGTTCAGGTTCAGGATCTTCGATCTCGGGACAACATTACAACGCTTTCAGAGGAATAGTGTACTTCAC<br>ATTTACTCACGGCTACAACCAGTACTCCGCATCGACTCGATTTGGTGTCTACCCTGACACTGGATTAATTGTCGACTCGAACGGCAAACCAA<br>TCTCCGGGACCGCGCCCCGCAACGCCTGCATCGACTATTCACCTCATGGTCCCACCGACGTT
SIX 11 (Sequences 5):<br>ATGATGTTCTCCAAAGCCATCTCAATTATCTCACTCCTTATAAGCACCAGCCACGCCATCAATATATGTTGCTCCTCCTTTGTTGGCCACACC<br>TGCACGAAAGATCAATATAATAACCACCGCCAGAATGTCATCTTGAACCAGATCATCGACAAAGACGGAATGAATTGCGTACGAAAGGGC<br>GCCGGGCCGGGGCGATGGACCACCAAAGGTGACTGGTCAGAGTGGTATGACTGTCAGCAGTGGAACGGGCCTGAGCAGCATCAGATCGAA<br>GTTGGAGAGTGTACTCTGTTTTGTGTCACACCGAGTGGCAATCTCAATAGACCCTGCAT
SIX 12 (Sequences 6):<br>ATACTGCTTCAAGTACAAACTACATTAGCCCAAGCGTCCAGTTGTCTCAGCGTTGGACCCAAAGGTATCAACAACCAGAATGCTTGTGTGT<br>GTGGAGGTCAATGCGTCATGGCAGACTTGGTCGTTGCTGGCAGAAAGATTTGCTGTGAATACACAGTTCAGATACAGGGTGGTTGGCCTGT<br>TCTTGCCAATAGCCATTGTGTTTACGGCTCTACTGGTGCCAACGGGGGTTCATGCTCTGGTAATCAAGTCAGCCTTGCTTGGTGGCACAACT<br>ACGAGCCGGAGGTGAAGAGCACAGATTCCAAATGCATTTTCGCGAAGCCCAAGCTATGCCACTC
SIX 14 (Sequences 7):<br>ATGCATATCGAGTATTTATTTCTACTTTTGTCCCAATGGGAGCTCTGTCTCAGCGTATCCTCGGCTGCCGCATGCCGAACGGATCCTTAAA<br>CCCAAGTCCAAATATATGTAACCAAGC

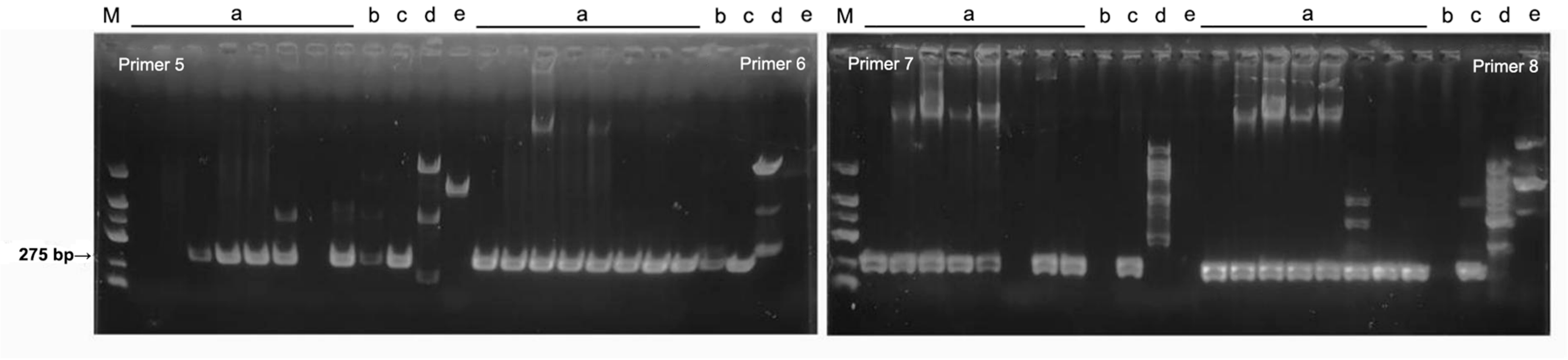

Supplementary Figure S1. Gel electrophoresis with 1% agarose of candidate primers for host-specialized strains of *Fusarium oxysporum* a: *F. oxysporum* saffron strains, *F. oxysporum* saffron strains (1: XHH2; 2: XHH16; 3: XHH35; 4: XHH47; 5: XHH56; 6: XHH66; 7: XHH67; 8: XHH76); b: *F. oxysporum* f. sp. *fragariae*; c: *F. oxysporum* f. sp. *lycopersici*; d: *F. oxysporum* f. sp. *niveum*; e: *F. oxysporum* f. sp. *melonis*; M: DNA Molecular Weight Standard Marker (100~2000 bp; Sangon Biotech, Shanghai, China)

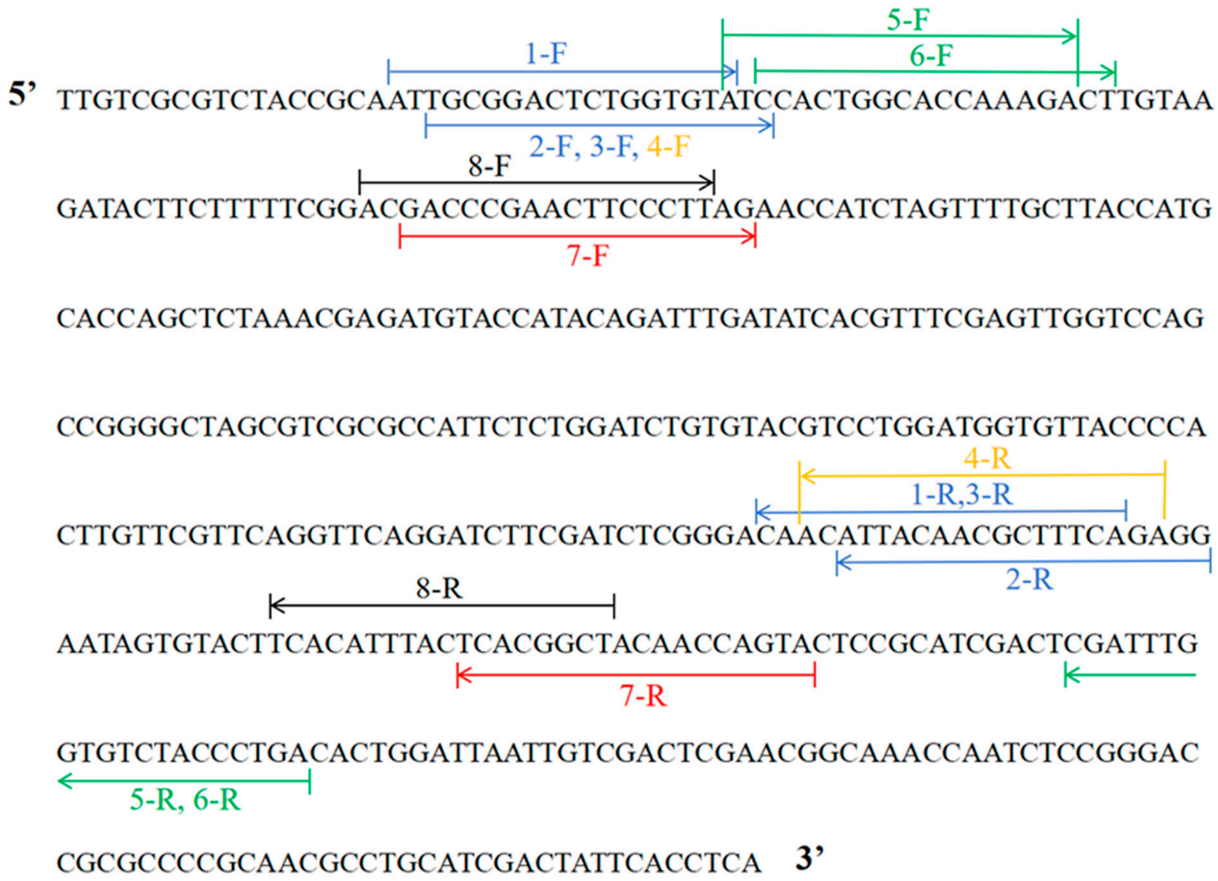

Supplementary Figure S2. Primers 1-8 have been shown to amplify the concrete regions of the *SIX 10* gene (partial sequences).
